# Intraspecific differences in long-term drought tolerance in perennial ryegrass

**DOI:** 10.1371/journal.pone.0194977

**Published:** 2018-04-04

**Authors:** Daliya Cyriac, Rainer W. Hofmann, Alan Stewart, P. Sathish, Christopher S. Winefield, Derrick J. Moot

**Affiliations:** 1 Agriculture and Life Sciences Division, Lincoln University, Lincoln, Christchurch, Canterbury, New Zealand; 2 PGG-Wrightson, Christchurch, Canterbury, New Zealand; 3 Indigenz Limited, Auckland, New Zealand; Estacion Experimental del Zaidin, SPAIN

## Abstract

*Lolium perenne* L. (perennial ryegrass) is the most important pasture grass species in temperate regions of the world. However, its growth is restricted in summer dry environments. Germplasm screening can be used to identify accessions or individual plants for incorporation into breeding programs for drought tolerance. We selected nine perennial ryegrass accessions from different global origins and from a range of climatic and environmental conditions. In addition, the perennial ryegrass cultivar ‘Grasslands Impact’ was chosen as a reference. The accessions were grown for 360 days in a controlled environment through six consecutive drought stress and recovery cycles. We observed intraspecific differences in drought stress responsiveness for shoot biomass and survival from the third stress cycle. An accession from Norway had 50% more shoot dry matter than the next best-performing accession after six drought cycles. Compared with the reference cultivar ‘Grasslands Impact’, shoot dry matter of the accession from Norway was more than seven times higher after six drought cycles, indicating superior performance of this ecotype under drought stress. Drought tolerance was characterized by osmotic adjustment and higher relative leaf water content at low soil moisture levels. Furthermore, the findings of this study identify solute potential as an early predictor of drought stress tolerance. These intraspecific differences can be used in breeding programs for the development of drought-tolerant perennial ryegrass cultivars.

## Introduction

A high proportion of milk and meat production in the world is supported by temperate grazed forage grasses dominated by perennial ryegrass [1]. This cool season, self-incompatible diploid (2n = 2x = 14) outcrossing species from the Poaceae family is native to Europe, Asia and northern Africa [2]. It is broadly adapted and cultivated as a forage species in the temperate regions of the world due to its high growth rate under fertile conditions. It is easy to establish and to manage with tolerance to animal treading and hard grazing and has comparatively high palatability and digestibility [1, 3]. However, perennial ryegrass fails to thrive under hot dry summer conditions [4–6], which limits its range of adaptation. Moisture limitation is the major environmental stress in agriculture worldwide and in a changing climate is expected to intensify in the future, which may constrain the yield and quality of perennial ryegrass [7].

Improvement of stress tolerance in perennial ryegrass is important for sustainable temperate forage production [8]. There are numerous tolerance mechanisms in plants under water deficit conditions [9]. Leaf responses to water deficit are initially characterised by a reduction in leaf length and width, followed by leaf abscission to reduce water loss via transpiration [10]. Osmotic adjustment may occur, whereby turgor potential is maintained to a degree by active accumulation of organic and inorganic solutes in cells. This reduces the osmotic potential and improves water retention in the cells under desiccation stress, which enables plants to continue to grow. Accumulation of compatible solutes protects enzymes and plasma membranes in the cytoplasm, whereas inorganic ions regulate the osmotic potential of the vacuole [9–11]. Osmotic adjustment under water deficit conditions benefits cell elongation and stomatal opening during the day. Several studies have linked osmotic adjustment to yield protection under drought stress [12–16]. A recent review of 12 crops reported osmotic adjustment as a prime adaptive trait under water deficit conditions [17].

Perennial ryegrass responses to soil moisture deficit have been extensively studied because of the economic importance of this species. Studies have investigated soil water extraction and water use [18, 19], the relationship between leaf ridging and desiccation stress [20], responses to sudden or gradual exposure to water deficits [21], the importance of spring management to improve drought tolerance [22] and recovery growth after severe soil moisture deficits [23]. The symbiotic relationship of endophytes and perennial ryegrass has been investigated in the context of drought stress, with some indications of possible stress-protective effects for perennial ryegrass by the endophyte [24–28]. Other studies used a transgenic approach to examine drought stress responses in perennial ryegrass [29–31]. Selection based on drought recovery has also been identified as a promising trait for breeding tolerance in this species [32]. However, effective drought tolerance in perennial ryegrass is difficult to achieve and detailed studies of physiological acclimatization to soil moisture deficits are needed, especially under long-term drought stress exposure.

Despite extensive research in perennial ryegrass breeding [33], currently there are no identified traits linked to drought tolerance in this species. Introgression with deeper rooting Mediterranean germplasm has been suggested as a means of introducing drought tolerance, to provide rapid regrowth in autumn into the winter season and high quality vegetative growth until late spring [34, 35]. However there is potential to identify suitable traits within ecotypes for plant breeders to develop drought-tolerant cultivars [36]. One difficulty is the need to screen large numbers of germplasm or ecotypes under multiple drought cycles, which requires time and resources. If this could be minimized by the identification of potential mechanisms early in the screening process, then faster progress to identify plants with superior performance under water deficit conditions could be made.

The aims of this study were to (i) identify germplasm with potential drought-tolerant phenotypes and (ii) to discern physiological attributes in such germplasm. This was done during multiple drought and irrigation cycles to examine the relative performance of phenotypes and physiological markers over time. Experimentally, accessions selected from a wide climatic and geographic range were established in two rhizotrons. Their agronomic and physiological performance were assessed during six drought cycles over 360 days.

## Materials and methods

### Germplasm accessions

Nine accessions from the Margot Forde Forage Germplasm Centre, New Zealand, were selected to evaluate germplasm from a range of climatic and environmental conditions. The accessions were A6889 (‘Otago/Southland', New Zealand), A6932 (‘Portugal’), A7798 ('France'), A14499 ('Turkey'), A14542 (‘Italy’), A15323 (‘Algeria'), A15334 (‘Cyprus'), A15369 (‘Tunisia'), A17183 (‘Norway'). In addition, the cultivar ‘Grasslands Impact’ (‘Impact’) was chosen as a reference. Detailed information about their acquisition and inclusion in the collection is given in the supporting information (S1 Appendix).

### Growth room and media

The experiment was conducted in a 5.0 X 2.4 m Conviron BDW120 growth chamber (Lincoln University Biotron facility, New Zealand) equipped with metal halide lamps (Model MS400W/HOR, Venture) and incandescent bulbs (100W, Philips). Lights were mounted above a clear Perspex barrier, and a downward airflow distribution system maintained the ambient (350–400 ppm) CO_2_ conditions. Underneath the growth room, two rhizotrons (107 cm length X 80 cm width) provided the soil environment for the plants in the growth room. The rhizotrons were filled with soil to form the top soil horizon “A” (24 cm) and the subsoil horizon “B” (24 cm) with a layer of sand (2 cm) under horizon “B”. A Templeton silt loam soil or Udic Haplustept [37] was sieved to remove stones and large pieces of plant material. It was then processed through a soil shredder. Horizon “A” was blended with sand in a 4:1 ratio (soil: sand by volume) and horizon “B” at a 5:2 ratio. Each horizon was recreated within each rhizotron to reflect bulk density equivalent to that found in the field. The resulting rhizotrons were placed in the controlled environment and the soil profiles were stabilized for more than 11 weeks by watering the rhizotrons until drainage occurred.

### Germination of accessions

To establish seedlings for transplanting into the rhizotrons, a single seed was sown into each cell plug of cell trays and these trays were watered and germinated in containment in the Biotron. After emergence, the seedlings were regularly watered to ensure there was no soil moisture deficit at any point in the seedling stage. Hoagland’s solution [38] was added at least every two weeks to ensure seedlings were never nutrient-deficient. The environmental conditions within the growth room during seedling and initial sward establishment were set to 15°C air temperature and 8°C soil temperature to obtain 99% seed germination [39]. The photoperiod was 16/8 h (day/night) and the relative humidity was maintained at 70%. A 30 min ramped twilight was applied either side of the daylight hours. The light intensity at plant level was 463 μmol.m^-2^.s^-1^ measured with a LI-COR® Radiation Sensor (LI-250A Light Meter, Turfschipper 114, 2292 JB Wateringen, Netherlands).

### Microsward establishment

Seedlings selected for transplanting were trimmed to a uniform height of 2mm and contained 2–3 tillers. They were then planted in microswards of six plants per swards in two rhizotrons in which each contained 20 plots. The perennial ryegrass accessions were arranged in randomized blocks. The rhizotrons accommodated 2 X 185 plants, including “Fill” plants, which provided four replicates across the two rhizotrons. Time Domain Reflectometry rods of 20 cm length were inserted to measure soil moisture content (Trace systems, Model 6050X1, Santa Barbara, California, USA).

### Drought cycles

**Fig 1:**
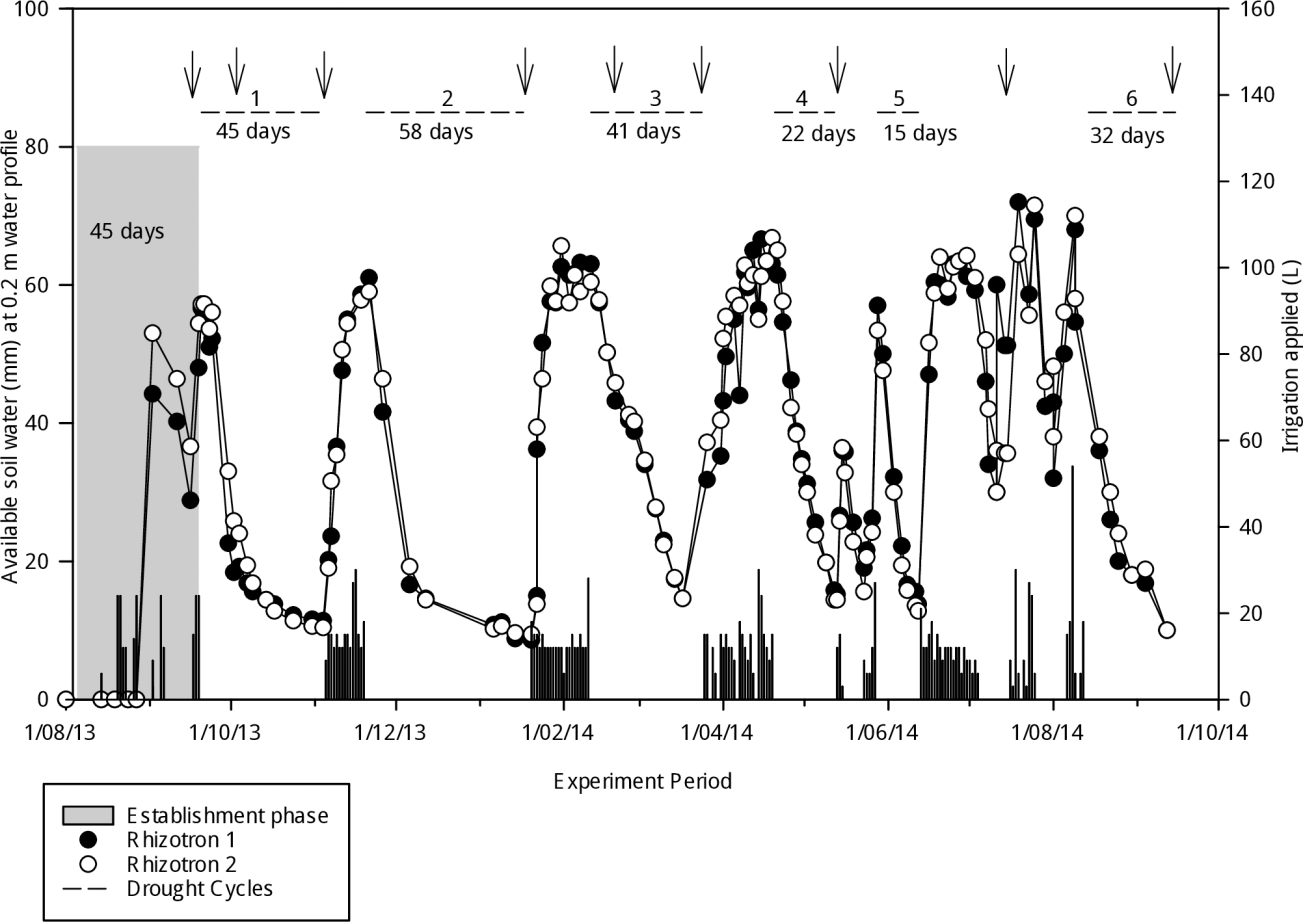
Description of the experiment, including soil moisture, irrigation application, drought cycles and harvest dates of microswards of perennial ryegrass accessions. Plants were grown in rhizotrons under controlled environmental conditions at Lincoln University, New Zealand. The shaded area represents the establishment phase; ‘l’ bars represent irrigation application rates. Drought cycles are shown as horizontal lines marked above the figure. Arrows (↓) represent harvest dates during and at the end of each stress cycle. Duration of establishment periods and stress cycles are shown below each stress cycle (—).

The rhizotrons were watered continuously during the 45 days of establishment phase to ensure vigorous seedling growth. The duration of the experiment, soil moisture profile, drought cycles, irrigation applied over the experiment period and harvest dates are shown in Fig 1 and a detailed summary in S1 Table. The study has six drought cycles. These are defined as the period between the last day of irrigation until the final shoot biomass harvest at the end of each regrowth period.

The duration of drought cycles ranged from 15 to 58 days. This reflects the variability of drought periods experienced in temperate regions such as the dairy growing areas of New Zealand, which rely on perennial ryegrass as their base pasture for grass-fed milk production [40]. In these regions the frequency of drought events lasting more than 30 days is expected to increase under future climate scenarios [41, 42]. Accordingly, the duration of drought stress was further extended to simulate such scenarios. The first harvest was carried out before onset of the first drought cycle on 18/9/2013. Drought Cycle 1 was initiated on 20/09/2013, with soil temperature set at 25°C and air temperature maintained at 15/25°C (night/day) in all six drought cycles (S1 Table). During the first cycle, the plants were harvested on 3/10/2013, after which the plants were not re-watered. A final harvest for Cycle 1 occurred on 4/11/2013 at approximately 5% V/V soil moisture content. The rhizotrons were then re-watered to field capacity (30% V/V) over 14 days. Drought Cycle 2 was commenced on 20/11/2013. Field capacity was identified as irrigating the rhizotrons with 3 Litres of water at 2–3 hour intervals until drainage of water through the bottom of the rhizotrons was detected. The re-growth phase lasted 58 days until harvest on 17/01/2014. Following this harvest, rhizotrons were left without watering for four days at ~4% V/V soil moisture. Then the rhizotrons were re-watered to field capacity over a 19-day period. Drought Cycle 3 was initiated on 11/2/2014 and ended on 24/3/2014 with a final harvest. The rhizotrons were re-watered to field capacity before initiating drought stress on 20/04/2014. Due to infestation with thrips, Cycle 4 was completed after 22 days with a shoot dry matter harvest on 12/05/2014. The rhizotrons were watered to field capacity before initiating the fifth drought cycle on 28/5/2014. This cycle was suspended after the rhizotrons were flooded due to the breakdown of the growth room facility on 13/6/2014. This caused a delay in initiating the next drought cycle, because it took a prolonged period to re-water the rhizotrons back to comparable soil moisture levels. The watering continued at a low rate until 14/08/2014 to maintain both rhizotrons at similar moisture levels. The final drought cycle, Cycle 6 was initiated on 14/08/2014 and was completed on 15/9/2014, which ended the experiment 360 days after establishment. Hoagland’s solution was added during every drought cycle to ensure adequate nutrient supply to the plants [38].

### Measurements

Dry matter from plants from each plot was harvested at the end of each drought cycle. The harvested plants were subsequently dried at 65°C to constant weight and total dry matter from each accession was calculated. Plant survival rate was also calculated at the end of each drought cycle, based on regrowth and dry matter production. Based on the criteria in Table 1, visual appearance of plants was used as a non-destructive measure to record the performance of plants from each accession during each drought cycle. The leaf extension rate was measured weekly on two tagged tillers of two different plants of each plot. Measurements were taken until the appearance of the ligule, which indicated that the leaf was fully expanded. The measurement was then shifted to the next newly emerging leaf on the same tiller. The marked tillers were replaced with new tillers in each irrigation period.

**Table 1:** Definitions of the plant grading scores used.

Grade	Definition
0	Dead
1	One or few live tillers
2	More live tillers but growth less than 25% of best performing plants
3	Plant growth 25%-50% of best performing plants
4	Plant growth 50%-75% of best performing plants
5	Plant growth 75%-100% of best performing plants

Two fully expanded leaves per plant were harvested to measure relative water content (RWC) and leaf area (LA) to minimise destructive measurements during the drought cycles. The harvest of leaves for different measurements coupled with senescence meant that there were fewer leaves towards the end of each drought cycle available for physiological measurements. Therefore, length and width of leaves harvested for RWC were measured each time and recorded to quantify the leaf area.

To calculate leaf area (LA), a fully expanded leaf from each plant was excised from the plant and the lamina area was scanned using a Leaf Area Meter (AM 300, 12 Spurling Works, Pindar Road, Hoddesdon, Herts EN110DB,UK). The values obtained from the leaf scanner were used to determine the relationship between leaf length and leaf width to estimate leaf area [43].

RWC was calculated using the following equation,

$$RWC=\left[\frac{\left(FM-DM\right)}{\left(SM-DM\right)}\right]x100$$ (1)

FM is leaf fresh Mass (g), DM is leaf dry mass (g), and SM is the saturated leaf mass (g) [44].

Leaf samples were collected for osmotic potential determination [45] were collected in 1.7 mL microfuge tubes. These were prepared by placing a metal mesh in the bottom of each tube. The leaves were placed on top of the metal mesh, so that the leaf sap could be collected at the bottom of the tube for measurements of cell sap osmolality (vapor pressure osmometer, WESCOR, Utah, USA). These microfuge tubes with leaf samples were snap frozen using liquid nitrogen. The tubes were subsequently spun at 12,200 g for 5 minutes to extract the leaf cell sap immediately upon thawing. Solute potential (Ψπ) was calculated from the osmolality of the leaf sap (mmol.kg-^1^) using the following equation,

Ψπ=−RTcj (2)

where, RT = -0.002437 m^3^ MPa.mol^-1^ at 20°C and cj is the total solute concentration or osmolality (mmol.kg^-1^) [46]

To understand the active accumulation of compatible solutes, adjusted solute potential was calculated. For this, the adjusted solute potential (*Ψ*_*S*_100) was estimated using the following equation,

$$\Psi s100=\Psi s\;\frac{RWC-0.1}{1-0.1}$$ (3)

where *Ψs* is solute potential and 0.1 is the estimated water content in apoplast tissue [47].

Endophyte detection was carried out following established methods [48]. Due to insufficient seed numbers, endophyte presence could not be tested in A6932 (Portugal) and A15334 (Cyprus). Seeds from the other accessions were sown in small black pots (7 cm x 7 cm x 8 cm). The plants were grown to the 3–4 tiller stage, then removed from the soil and the main tiller was cut from the base. Necrotic sheaths were carefully removed from the main tiller. The tillers were cut transversely and the cut end was pressed onto a nitrocellulose membrane (NCM) (0.45 mm) [48]. This results in a circular moist mark on the NCM. The blotted paper was stored at 4°C until processing. Ryegrass tillers of known endophyte status were blotted as positive and negative controls. The NCM was developed and endophyte detection was carried out as described previously [48].

### Statistical analysis

Dry matter production from repeated harvesting of the same plants over the period of the experiment was analysed by REML (Restricted Maximum Likelihood) in Genstat Version Release 16.1 (Copyright 2013, VSN International Ltd.) to examine the interaction of accessions and harvest dates over time. Furthermore, dry matter production was analysed with REML using residuals from the previous harvest as the covariate to examine the contribution of plant death to dry matter production and the variation among residuals. Subsequently, total accumulated dry matter and dry matter production from individual harvests were analysed by one-way Anova in randomized blocks to compare the performance of each accession individually at each time point. Best subset regression was carried out using the Minitab 17 Statistical Software (2010), with the area as the response variable using length and width as predictors to generate leaf area. All physiology measurements were analysed by one-way Anova, and Fisher’s protected LSD was used for means separation when significant. Two-way Anova using accessions and date was used to analyse adjusted solute potential from the final drought cycle. As the drought progressed, some accessions died. Accessions with only 0, 1 or 2 remaining live replicates were removed from the analysis at each time point. SEMs from one-way Anova analyses are shown where differences among accessions (α = 0.05) were detected at a given date, and the highest SEM across dates is shown in figures where no date-specific differences among accessions were detected in the Anova. Relationships between parameters were tested using regression analysis and Pearson correlation coefficients in Minitab 17 (2010).

## Results

### Shoot dry matter accumulation

**Fig 2:**
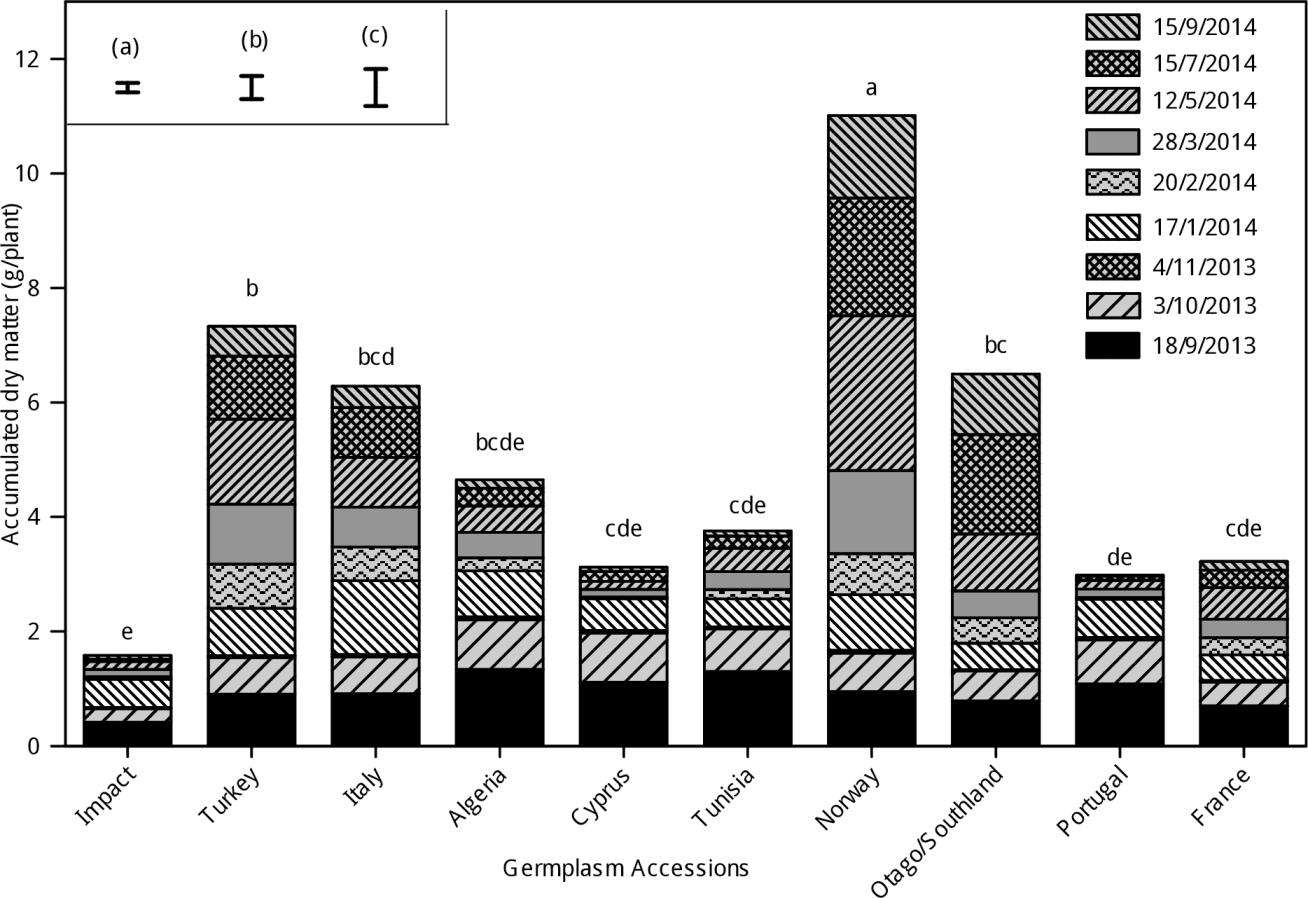
Shoot dry matter accumulation of 10 perennial ryegrass accessions at nine harvest dates. Error bars are LSDs for accessions (a), harvest date (b) and accessions x harvest date (c) from REML analysis. Bars with letters in common are not different (α = 0.05) in the Anova.

There was an interaction (P<0.001) between accessions and harvest date for plant dry matter production (Fig 2). Analysis using residuals from the previous harvest as a co-variate showed differences in dry matter production on 3/10/2013 and 28/03/2014 to 15/9/2014 (P<0.001). The accession ‘Norway’ had the highest (P<0.001) mean shoot biomass accumulation (11 ± 1.20 g/plant) after six stress cycles, whereas ‘Impact’ only had 1.5± 1.20 g/plant. The dry matter accumulation of 'Turkey’ was second-highest (7.3 ± 1.20 g/plant, Fig 2).

### Plant survival

**Fig 3:**
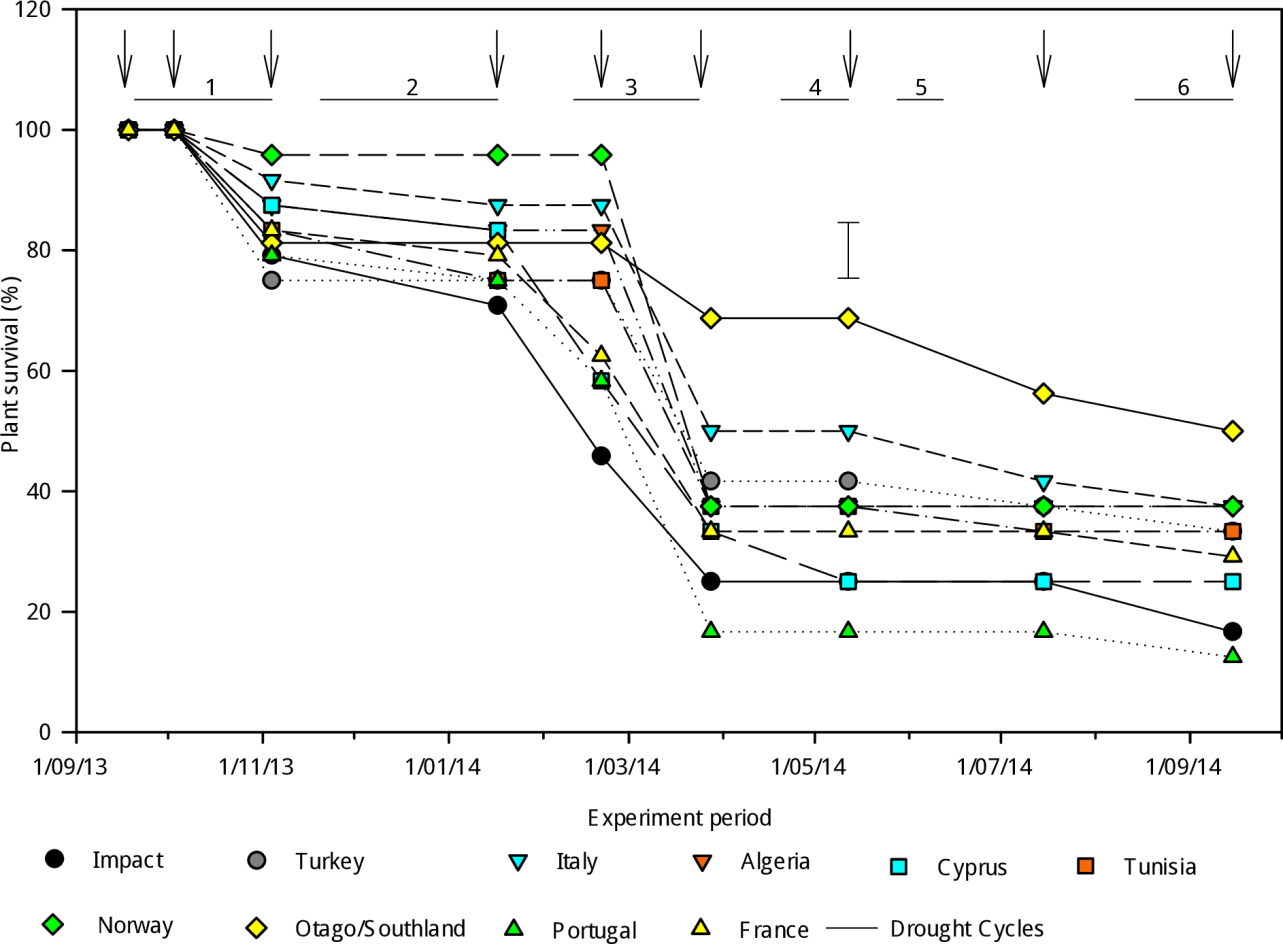
Plant survival of perennial ryegrass accessions at the end of each drought cycle. Error bars for plant survival are SEMs from one-way Anova in randomized blocks and are shown where differences (α = 0.05) were detected at a given date. ↓ indicates shoot harvest date.

Plant survival (Fig 3) after the first two harvests, before imposing drought stress at Cycle 1, was 100% across all accessions. No difference in plant survival rate was observed after Cycle 1 (P = 0.45), Cycle 2 (P = 0.71) and Cycle 3 (P = 0.27). However a trend for differences in plant survival was observed after Cycle 3 (P = 0.06) and differences (P<0.05) were observed during Cycle 4, where the ‘Otago/Southland’ accession (68.8 ± 9.23%) had the highest survival rate. The lowest survival rate was observed in the ‘Portugal’ (16.67 ± 9.23%) accession. The plant survival rate of the ‘Norway’ accession remained stable from Cycle 3 (37.5 ± 10.64%) to the end of Cycle 6 (37.5 ± 8.95%).

### Physiology

**Fig 4:**
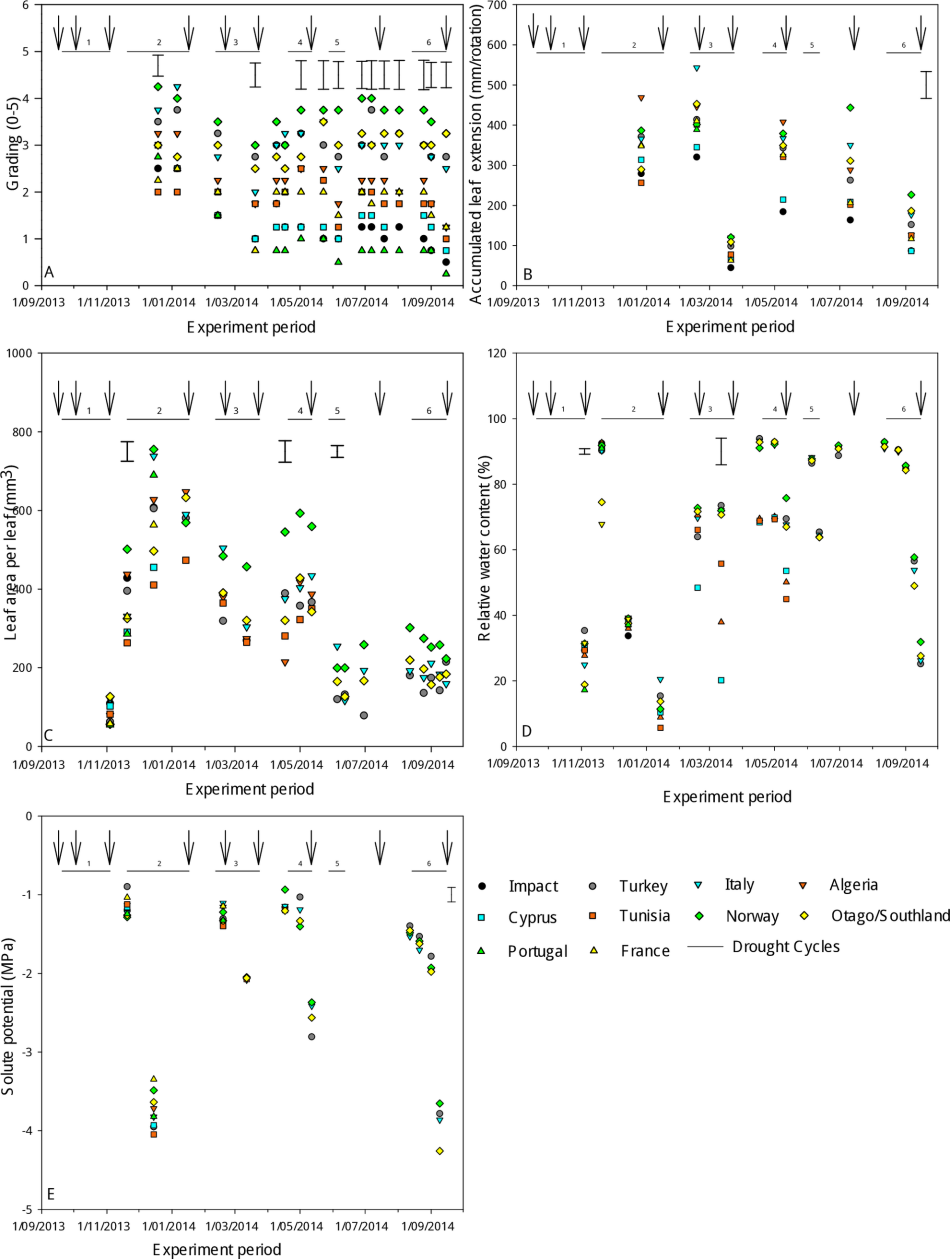
Fig 4A: Plant grades (growth scores), Fig 4B: Accumulated leaf extension (mm/rotation), Fig 4C: Leaf size (area per leaf), Fig 4D: Relative water content (%), and Fig 4E: Solute potential (MPa) of perennial ryegrass accessions during six drought cycles. Error bars are SEMs for accessions at a given date (Fig 4A, 4C and 4D) or across harvest dates (Fig 4B and 4E) from one-way Anova. ↓ indicates shoot harvest dates.

Initial grading (Table 1 and Fig 4) at the middle of Cycle 2 showed ‘Norway’ had the highest (P<0.05) size score (4.3 ± 0.44) and ‘Tunisia’ the lowest (2 ± 0.44). The size grades of the accessions were not different (P = 0.13) at the beginning of Cycle 2, whereas ‘Norway’ had a high score (3± 0.51) by the end of Cycle 2. The highest (P<0.05) grading values during Cycles 4, 5 and 6 were shown by the ‘Norway’, ‘Otago/Southland’, ‘Turkey’ and ‘Italy’ accessions. At the end of Cycle 6, ‘Norway’, ‘Otago/Southland’, ‘Turkey’ and ‘Italy’ had the highest size scores (p<0.001) and ‘Tunisia’, ‘Cyprus’, ‘Impact’, and ‘Portugal’ the lowest (P<0.01, 0.25 to 1 ± 0.54).

By the end of Cycle 5, ‘Norway’ tended to have the longest (P = 0.07) leaf extension (443 ± 49.8) and ‘Impact’ the shortest (Fig 4B). This pattern continued through Cycle 6 with an indication of longer (P = 0.06) leaf extension (226 ± 25.6) also shown for ‘Norway’.

The highest adjusted R^2^ (R^2^_adj_ = 0.82) for leaf area per leaf was generated using length as a predictor (Eq 4). The constant (-147) resulted in negative leaf area when leaf length was less than 46 mm. However, this equation was accepted because the adjusted R^2^ decreased (R^2^_adj_ = 0.75) when the intercept was forced through the origin.

Area=−147+3.2(±0.27)*Length (4)

The leaf area of these accessions were not different (P = 0.35) at the end of Cycle 1 (Fig 4C). ‘Norway’ had the largest leaf area (502 ± 49.8 mm^2^, P<0.05) at the beginning of Cycle 2 and ‘Algeria’, ‘Impact’ and ‘Turkey’ were not different to ‘Norway’. At the end of Cycle 3, the leaf areas of the accessions ranged from 265 to 457 ± 43.6 mm^3^ and there was a trend (P = 0.055) for ‘Norway’ to have the largest area. At the beginning of Cycle 4, ‘Norway’ had the largest (P<0.05) leaf area of 545 ± 55 mm^3^, whereas ‘Turkey’ had the largest leaf area of 254 ± 21.2 mm^3^ at the beginning of Cycle 5. There was no difference in leaf area during Cycle 6 among the surviving accessions.

‘Turkey’ had the highest (P<0.001) RWC (35 ± 1.7%) at the end of Cycle 1 and accessions ‘France’, ‘Norway’ and ‘Algeria’ were not different to Turkey (Fig 4D). ‘Portugal’ and ‘Otago/Southland’ had the lowest RWC (18% and 17 ± 1.7%). During Cycle 2, RWC was measured at three time points under progressive drought, and accessions were not different (P = 0.87) from each other. At the end of Cycle 3, the accessions that showed the highest (P<0.05) RWC were ‘Algeria’, ‘Norway’, ‘Turkey’, ‘Otago/Southland’ and ‘Italy’ and this ranged from 70% to 75 ± 8%. ‘Cyprus’ had the lowest RWC (20 ± 8%) at the end of Cycle 3. At the end of Cycle 4, RWC ranged from 54% in Cyprus to 76 ± 7% in ‘Norway’. In Cycles 5 and 6, the only surviving accessions were ‘Norway’, ‘Otago/Southland’, ‘Turkey’ and ‘Italy’ and these were not different to each other in their RWC (P = 0.96) at any time point.

Solute potential was not different (P = 0.75) among accessions during Cycle 2 (Fig 4E). ‘Norway’, ‘Italy’, ‘Turkey’ and ‘Otago Southland’ had similar solute potentials from Cycles 3 to 6. The solute potential ranged from (-1.6 and -1.3 ± 0.05 MPa) at the beginning of Cycle 6. The solute potential at the end of Cycle 6 ranged between -4.2 and -3.7 ± 0.17 MPa among the surviving accessions.

### Adjusted solute potential

**Fig 5:**
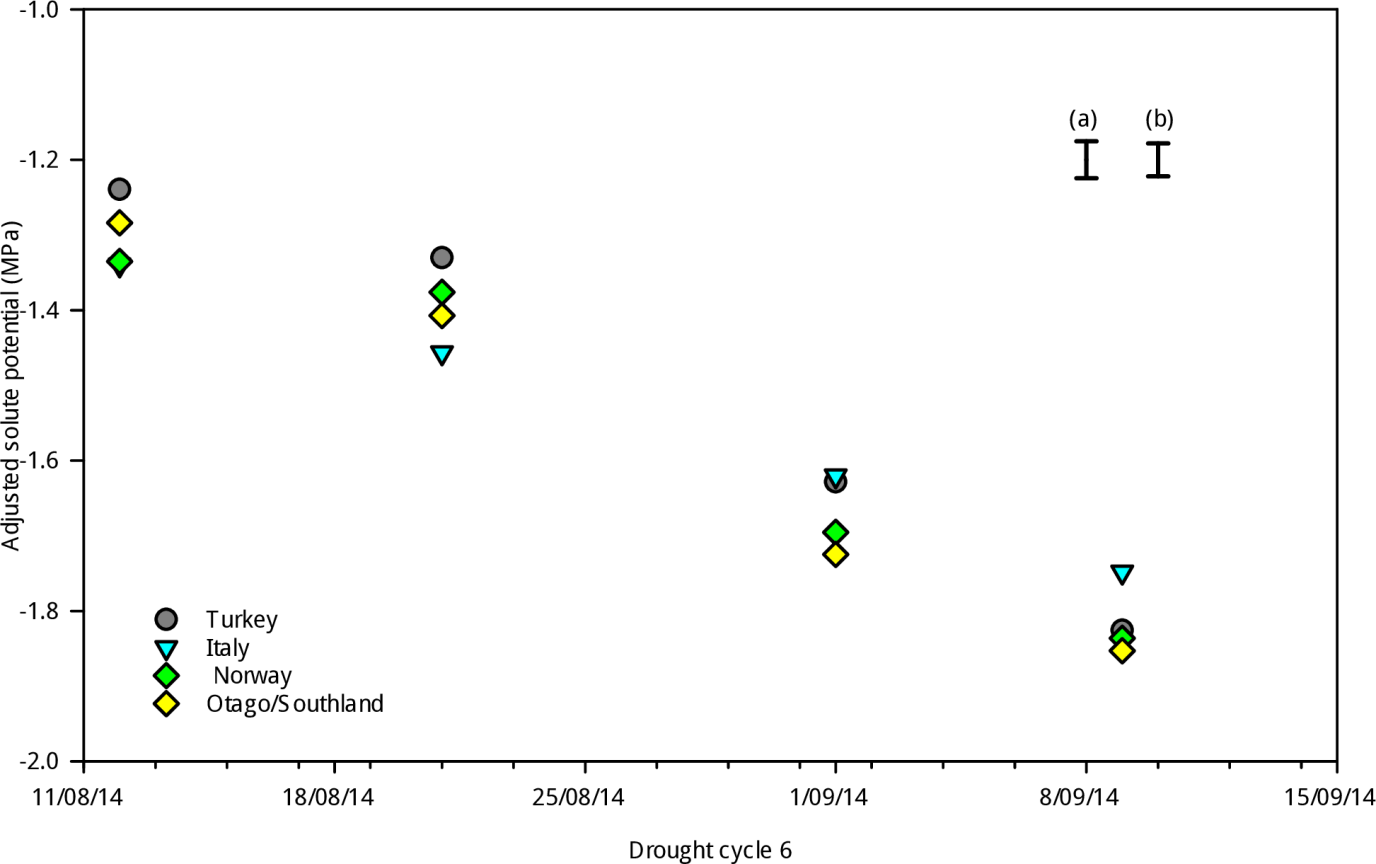
Adjusted solute potential of surviving perennial ryegrass accessions in the final drought cycle. Error bars are SEMs for date (a) accession (b) from two-way Anova.

The adjusted solute potential of the surviving accessions in Cycle 6 became more negative (P<0.001) as the drought stress progressed from 12/8/2014 to 9/9/2014. There was no difference (P = 0.35) in this response among the accessions (Fig 5).

### Relationships

**Fig 6:**
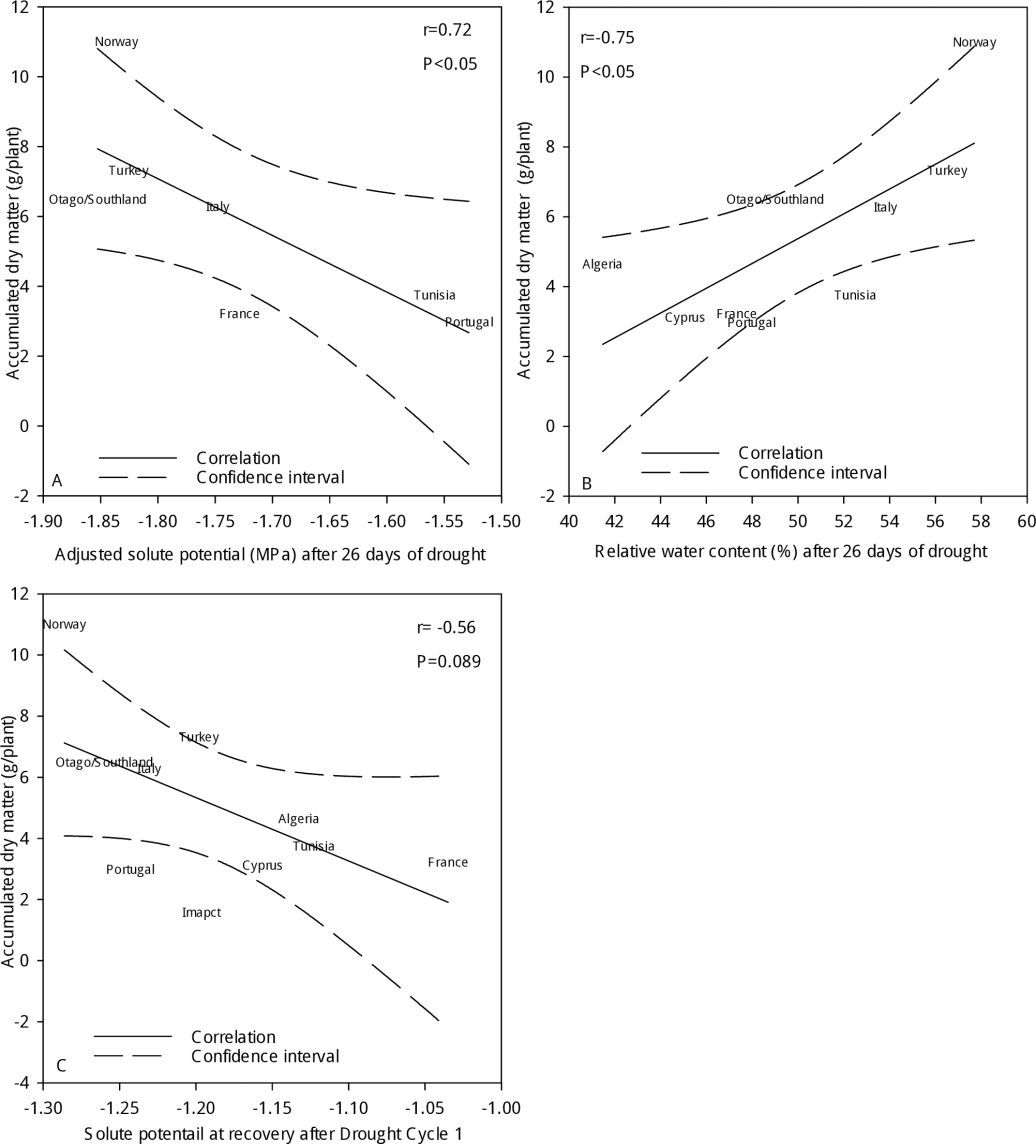
**Relationships of accumulated shoot dry matter of perennial ryegrass accessions at the end of six drought cycles** with relative water content (Fig 6A) and with adjusted solute potential after 26 days of drought in Cycle 6 (Fig 6B), as well as with solute potential after recovery from Cycle 1 (Fig 6C).

There was an association between accumulated shoot dry matter at the end of Cycle 6 and relative water content (r = 0.72, P<0.0.5) after 26 days of drought in Cycle 6 (Fig 6A). Accumulated shoot biomass after six drought cycles was inversely related to adjusted solute potential (r = -0.75, P<0.05) after 26 days of drought in Cycle 6 (Fig 6B). Furthermore, there was a tentative relationship (r = -0.56, P = 0.08) of the amount of dry matter that had accumulated after six drought cycles with solute potential after the recovery of the plants from Cycle 1 (Fig 6C).

## Discussion

Accessions of perennial ryegrass selected from a wide climatic and geographical range were successfully established in two rhizotrons, which enabled six drought cycles to be imposed to evaluate plant performance over 360 days. The extent of the desiccation stress and range of plant material chosen resulted in the identification of ecotypes with superior survival. This material provides opportunities for plant breeders to improve drought tolerance in perennial ryegrass. Similarly, ecotype germplasm has been used in the development of drought-tolerant intraspecific pair crosses of white clover, which is frequently grown as companion species with perennial ryegrass [49]. Wild or landrace germplasm has also been proposed for the improvement of winter hardiness in perennial ryegrass [50]. Future studies could investigate the genetic distances between the accessions used.

By the end of the experiment, the highest total shoot dry matter accumulated in the accession from ‘Norway’ (A17183). As expected, the accessions with high shoot dry matter accumulation at the end of the experiment were those with the highest survival rate and plant grades. Plant survival represented the number of regenerated plants, but this does not indicate their vigour or morphological status. ‘Norway’ had the highest yield but lower survival than the New Zealand accession ‘Otago/Southland’ at the end of Cycle 6. However its growth was more vigorous as indicated by higher plant size grades (Fig 4A), leaf extension (Fig 4B) and leaf area (Fig 4C) from drought cycle 3 onwards. At the end of Cycle 2, RWC was low (Fig 4D) in all accessions, indicating that the severity of water deficit used in this experiment was high when compared with other studies [26, 51, 52]. The severe drought stress experienced in Cycle 2 and duration of re-watering are characteristic of drought stress post-grazing. This appeared to contribute to the death of plants and caused a delay in the recovery phase of Cycle 3 in which plant loss was also high. It is possible that dry matter accumulation of ‘Norway’ benefitted from the shorter drought cycles that inadvertently occurred in Cycles 4 and 5 (Fig 2) which lasted 22 and 15 days, respectively. However, the availability of water was similar for all the accessions and it is notable that ‘Norway’ had comparatively longer leaves and more vigorous tiller regeneration. This is consistent with previous research for tiller survival ranking through winter, where ‘Norway’ had a similar ranking to the top eight superior performing accessions [50]. The dry matter accumulation of ‘Norway’ was also characterised by generally high levels of RWC (Fig 4D) and of solute accumulation (Fig 5) during the drought cycles. Taken together, this suggests morphological and physiological acclimation to drought in ‘Norway’. High RWC and unchanged solute potential indicates the absence of osmotic stress during the initial exposure to water deficit in ‘Norway’. High RWC levels from drought cycles 3 to 6 enabled ‘Norway’ to maintain a degree of leaf growth and protection of cellular components.

Osmotic adjustment is a drought tolerance strategy in plants in which active accumulation of compatible solutes decreases the solute potential and therefore promotes water uptake [26, 45]. The solute potential values (Fig 4E) after 30 days of water withdrawal were consistent with solute potential values observed elsewhere after 20 to 40 days of water withdrawal in perennial ryegrass [53]. At that stage, the leaf water content (Fig 4D) was only ~40% in most accessions. The results suggest that the surviving accessions preserved their meristems and the integrity of metabolic functions by tolerating decreased leaf water content via accumulation of compatible solutes that acted as osmolytes and osmoprotectants and supported their recovery after water was withdrawn. This is reflected by the reduced adjusted solute potentials in these accessions (Fig 5). The accumulation of compatible solutes protects the protein-synthesizing machinery against the damage caused by water withdrawal. This in turn helps to repair the stress induced damage more efficiently and rapidly than the rate of damage occurring [54].

Dry matter accumulation was related to relative water content (Fig 6A) and adjusted solute potential (Fig 6B), illustrating that sustained drought tolerance in perennial ryegrass was effected by osmotic adjustment. Osmotic adjustment can be the prime adaptive trait that supports plant yield under soil water deficit conditions [17]. This is further substantiated by the observation that the drought-resistant perennial ryegrass accessions were able to retain more water in their leaves during periods of water deficit (Fig 6B). These relationships suggest that reduced adjusted solute potential could be a potential screening method to identify high yielding ecotypes in the field after experiencing drought stress during summer. A further relationship (Fig 6C) gave an indication that stress responsiveness after six drought cycles was linked to plant solute potential after Cycle 1 highlighting solute potential as possible early predictor of long-term drought tolerance. This method could thus be incorporated into germplasm screens for drought tolerance, using exposure to a single drought stress period.

Perennial ryegrass accessions sourced from the Mediterranean (‘Italy’, ‘Portugal’, ‘Turkey’, ‘Tunisia’ and ‘Algeria’) experience in their natural habitat mild winters and dry and warm summers [55]. In contrast to the Mediterranean zone, the significantly colder provenance of the ‘Norway’ accession includes frozen ground for extended periods of the year [56]. These conditions also mean that plants experience freezing-induced dehydration [57]. The Norwegian accession was able to survive and remain relatively productive in this study, and this could be due to cross talk between stress signalling pathways, providing cross-tolerance for improved physiological function to survive and maintain relative productivity under sustained drought exposure [58–61]. Finally, the involvement of the endophyte symbiosis in drought tolerance [24–26, 62–64] deserves consideration. Analysis of the endophyte symbiosis of these accessions (S2 Table) showed no infection in ‘Norway’, which gives confidence to exclude endophyte symbiosis as a potential drought tolerance mechanism in ‘Norway’. One possible explanation for our findings could be that ‘Norway’ is late flowering and thus could have invested more carbon into vegetative growth compared to the other accessions. However, in this experiment, flowering was prevented in all accessions by removal of flowering buds. Furthermore, and similar to conditions under grazing in the field, the plant material was removed by cutting at regular intervals, thus bringing the accessions to a common starting point at each treatment cycle [65].

## Conclusion

Differential plant desiccation responses only became apparent after several drought cycles. If the study had finished after the first few cycles, such differences would not have been measured. The results from this work suggest that analysing accessions under multiple drought cycles for an extended period is required to identify drought-tolerant phenotypes. Further, screening ecotypes from cold climate backgrounds could potentially identify other drought-tolerant perennial ryegrass phenotypes. Collectively, ‘Norway’ showed highest productivity from drought Cycles 3 to 6, which highlights its superior performance under repeated drought stress due to its plant survival rate, leaf extension, RWC and osmotic potential. These results suggest merit for using such ecotype accessions as potential candidates for further investigation in breeding programmes towards drought tolerance in perennial ryegrass. Further, RWC and adjusted solute potential were identified as particularly relevant predictors of plant performance under drought.

## Supporting information

S1 Appendix Background information of germplasm accessions. (PDF)

S2 Appendix Photographs of rhizotron. (PDF)

S1 Table Summary of treatments and periods of each treatment under the study. (PDF)

S2 Table Results from endophyte screening from the seedlings generated from the available seeds. (PDF)

## References

[1] Foito A, Byrne SL, Shepherd T, Stewart D, Barth S. Transcriptional and metabolic profiles of *Lolium perenne* L. genotypes in response to a PEG-induced water stress. Plant Biotechnology Journal. 2009;7(8):719–32. doi: 10.1111/j.1467-7652.2009.00437.x 19702648 10.1111/j.1467-7652.2009.00437.x

[2] Cornish MA, Hayward MD, Lawrence MJ. Self-incompatibility in ryegrass. Heredity. 1979;43(1):95–106.

[3] Easton H, Stewart A, Kerr G. Ryegrass in pastures–breeding for resilience. Pasture Persistence. 2011;15:139–48.

[4] Hunt WF, Easton HS. Fifty years of ryegrass research in New Zealand. Proceedings of the New Zealand Grassland Association. 1989;50:11–23.

[5] Charlton J, Stewart A, editors. Pasture species and cultivars used in New Zealand-a list. Proceedings of the New Zealand Grassland Association; 1999; 147–166.

[6] Liu S, Jiang Y. Identification of differentially expressed genes under drought stress in perennial ryegrass. Physiologia Plantarum. 2010;139(4):375–87. doi: 10.1111/j.1399-3054.2010.01374.x 20444191 10.1111/j.1399-3054.2010.01374.x

[7] Mir RR, Zaman-Allah M, Sreenivasulu N, Trethowan R, Varshney RK. Integrated genomics, physiology and breeding approaches for improving drought tolerance in crops. Theoretical and Applied Genetics. 2012;125(4):625–45. doi: 10.1007/s00122-012-1904-9 22696006 10.1007/s00122-012-1904-9 PMC3405239

[8] Cui Y, Wang J, Wang X, Jiang Y. Phenotypic and genotypic diversity for drought tolerance among and within perennial ryegrass accessions. HortScience. 2015;50(8):1148–54.

[9] Fang Y, Xiong L. General mechanisms of drought response and their application in drought resistance improvement in plants. Cellular and Molecular Life Sciences. 2015;72(4):673–89. doi: 10.1007/s00018-014-1767-0 25336153 10.1007/s00018-014-1767-0 PMC11113132

[10] Munné-Bosch S, Alegre L. Die and let live: leaf senescence contributes to plant survival under drought stress. Functional Plant Biology. 2004;31(3):203–16. 10.1071/FP03236 32688892

[11] Morgan JM. Osmoregulation and water stress in higher plants. Annual Review of Plant Physiology. 1984;35(1):299–319.

[12] Thomas H. Osmotic adjustment in *Lolium perenne*; its heritability and the nature of solute accumulation. Annals of Botany. 1990;66(5):521–30.

[13] Thomas H. Physiological responses to drought of *Lolium perenne* L.: measurement of, and genetic variation in, water potential, solute potential, elasticity and cell hydration. Journal of Experimental Botany. 1987;38(1):115–25.

[14] Thomas H. Accumulation and consumption of solutes in swards of *Lolium perenne* during drought and after rewatering. New Phytologist. 1991;118(1):35–48.

[15] Thomas H, Evans C. Effects of divergent selection for osmotic adjustment on water relations and growth of plants of *Lolium perenne*. Annals of Botany. 1989;64(5):581–7.

[16] Jongdee B, Fukai S, Cooper M. Leaf water potential and osmotic adjustment as physiological traits to improve drought tolerance in rice. Field Crops Research. 2002;76(2):153–63.

[17] Blum A. Osmotic adjustment is a prime drought stress adaptive engine in support of plant production. Plant, Cell & Environment. 2016;40(1):4–10. 10.1111/pce.12800 27417527

[18] Garwood E, Williams T. Soil water use and growth of a grass sward. The Journal of Agricultural Science. 1967;68(02):281–92.

[19] Garwood E, Williams T. Growth, water use and nutrient uptake from the subsoil by grass swards. The Journal of Agricultural Science. 1967;69(01):125–30.

[20] Wilson D. Stomatal diffusion resistances and leaf growth during droughting of *Lolium perenne* plants selected for contrasting epidermal ridging. Annals of Applied Biology. 1975;79(1):83–94.

[21] Jones M, Leafe E, Stiles W. Water stress in field‐grown perennial ryegrass. Annals of Applied Biology. 1980;96(1):87–101.

[22] Korte C, Chu A, editors. Some effects of drought on perennial ryegrass swards. Proceedings of the New Zealand Grassland Association; 1983.

[23] Norris I, Thomas H. The effects of cutting on regrowth of perennial ryegrass selections exposed to drought conditions. The Journal of Agricultural Science. 1982;99(3):547–53.

[24] Hesse U, Schöberlein W, Wittenmayer L, Förster K, Warnstorff K, Diepenbrock W, et al Effects of Neotyphodium endophytes on growth, reproduction and drought‐stress tolerance of three *Lolium perenne* L. genotypes. Grass and Forage Science. 2003;58(4):407–15.

[25] He L, Matthew C, Jones C, Hatier J. Productivity in simulated drought and post-drought recovery of eight ryegrass cultivars and a tall fescue cultivar with and without *Epichloë* endophyte. Crop and Pasture Science. 2017;68(2):176–87.

[26] He L, Hatier JHB, Matthew C. Drought tolerance of two perennial ryegrass cultivars with and without AR37 endophyte. New Zealand Journal of Agricultural Research. 2017:1–16.

[27] Kane KH. Effects of endophyte infection on drought stress tolerance of *Lolium perenne* accessions from the Mediterranean region. Environmental and Experimental Botany. 2011;71(3):337–44.

[28] Cheplick GP. Recovery from drought stress in *Lolium perenne* (Poaceae): Are fungal endophytes detrimental. American Journal of Botany. 2004;91(12):1960–8. doi: 10.3732/ajb.91.12.1960 21652344 10.3732/ajb.91.12.1960

[29] Han L, Li X, Liu J, Zeng H. Drought-tolerant transgenic perennial ryegrass (*Lolium perenne* L.) obtained via particle bombardment gene transformation of CBF3/DREB1A gene. Acta Horticulturae. 2008;783:273.

[30] Zhang Z-X, Zheng Y-Z. Overexpression of nicotianamine synthase (NAS) gene results in enhanced drought tolerance in perennial ryegrass. Biotechnology & Biotechnological Equipment. 2008;22(4):938–41.

[31] Patel M, Milla-Lewis S, Zhang W, Templeton K, Reynolds WC, Richardson K, et al Overexpression of ubiquitin-like LpHUB1 gene confers drought tolerance in perennial ryegrass. Plant Biotechnology Journal. 2015;13(5):689–99. doi: 10.1111/pbi.12291 25487628 10.1111/pbi.12291

[32] Westermeier P, Hartmann S, editors. Varying growth behavior of Lolium perenne L. clones under drought conditions and after rewatering. The multiple roles of grassland in the European bioeconomy Proceedings of the 26th General Meeting of the European Grassland Federation, 4–8 September 2016; 2016; Trondheim, Norway,: NIBIO.

[33] Brummer EC, Bouton JH, Casier MD, McCaslin MH, Waldron BL. Grasses and legumes: genetics and plant breeding In: Wedin WF, Fales S. L, editor: Grassland: Quietness and Strength for a new American Agriculture. Madison, Wisconsin: ASA-CSSS-SSSA; 2009 p. 157–71.

[34] Lelièvre F, Volaire F. Current and potential development of perennial grasses in rainfed Mediterranean farming systems. Crop Science. 2009;49(6):2371–8.

[35] Matthew C, van der Linden A, Hussain S, Easton HS, Hatier JHB, Horne DJ. Which way forward in the quest for drought tolerance in perennial ryegrass? Proceedings of the New Zealand Grassland. Proceedings of the New Zealand Grassland Association 2012. p. 195–200.

[36] Humphreys M, editor The contribution of conventional plant breeding to forage crop improvement. Proceedings 18th International Grassland Congress (Association Management Centre: Calgary, Canada); 1997.

[37] Cox JE. Soils and agriculture of part Paparua County, Canterbury, New Zealand Soil Bureau bulletin 1978;34.

[38] Hoagland DR, Arnon DI. The water-culture method for growing plants without soil Circular California Agricultural Experiment Station 1950;347(2nd edit).

[39] Moot D, Scott W, Roy A, Nicholls A. Base temperature and thermal time requirements for germination and emergence of temperate pasture species. New Zealand Journal of Agricultural Research. 2000;43(1):15–25.

[40] Verkerk G. Pasture-based dairying: challenges and rewards for New Zealand producers. Theriogenology. 2003;59(2):553–61. 12499003 10.1016/s0093-691x(02)01239-6

[41] Chappel PR. The climate and weather of Waikato NIWA Science and Technology ND;61(2 edition):1–40.

[42] Salinger J, editor Climate reality-actual and expected Legumes for Dryland Pastures 2003; Lincoln University.

[43] Kosgey J. Elucidating the physiological mechanism of 'stay green' in maize hybrids-crop growth processes and nitrogen economy: electronic, scholarly journal PhD Thesis, Lincoln University 2011, Avalilable from: http://researcharchive.lincoln.ac.nz/handle/10182/4150

[44] Shepherd W. Temperature effects in determinations of leaf relative water content. Grass and Forage Science. 1977;32(4):225–6.

[45] Esperón‐Rodríguez M, Curran TJ, Camac JS, Hofmann RW, Correa‐Metrio A, Barradas VL. Correlation of drought traits and the predictability of osmotic potential at full leaf turgor in vegetation from New Zealand. Austral Ecology. 2018.

[46] Blum A. Osmotic adjustment and growth of barley genotypes under drought stress. Crop Science. 1989;29(1):230–3.

[47] Wilson J, Fisher M, Schulze E-D, Dolby G, Ludlow M. Comparison between pressure-volume and dewpoint-hygrometry techniques for determining the water relations characteristics of grass and legume leaves. Oecologia. 1979;41(1):77–88. doi: 10.1007/BF00344838 28310361 10.1007/BF00344838

[48] Simpson WR, Schmid J, Singh J, Faville MJ, Johnson RD. A morphological change in the fungal symbiont *Neotyphodium lolii* induces dwarfing in its host plant *Lolium perenne*. Fungal Biology. 2012;116(2):234–40. doi: 10.1016/j.funbio.2011.11.006 22289769 10.1016/j.funbio.2011.11.006

[49] Ballizany WL, Hofmann RW, Jahufer MZZ, Barrett BA. Multivariate associations of flavonoid and biomass accumulation in white clover (*Trifolium repens*) under drought. Functional Plant Biology. 2012;39(2):167–77. 10.1071/FP11193 32480771

[50] Hulke, Eric Watkins, Donald Wyse, Ehlke N. Winterhardiness and turf quality of accessions of perennial ryegrass (Lolium perenne L.) from public collections. Crop Science 2007;47:1596–608.

[51] Liu J, Xie X, Du J, Sun J, Bai X. Effects of simultaneous drought and heat stress on Kentucky bluegrass. Scientia Horticulturae. 2008;115(2):190–5.

[52] Mohammadi MHS, Etemadi N, Arab MM, Aalifar M, Arab M, Pessarakli M. Molecular and physiological responses of Iranian Perennial ryegrass as affected by Trinexapac ethyl, Paclobutrazol and Abscisic acid under drought stress. Plant Physiology and Biochemistry. 2017;111:129–43. doi: 10.1016/j.plaphy.2016.11.014 27915174 10.1016/j.plaphy.2016.11.014

[53] Volaire F, Thomas H, Bertagne N, Bourgeois E, Gautier M-F, Lelievre F. Survival and recovery of perennial forage grasses under prolonged Mediterranean drought: II. Water status, solute accumulation, abscisic acid concentration and accumulation of dehydrin transcripts in bases of immature leaves. New Phytologist. 1998;140(3):451–60. 10.1111/j.1469-8137.1998.00287.x 33862875

[54] Chen TH, Murata N. Enhancement of tolerance of abiotic stress by metabolic engineering of betaines and other compatible solutes. Current Opinion in Plant Biology. 2002;5(3):250–7. 11960744 10.1016/s1369-5266(02)00255-8

[55] Bouma E. Development of comparable agro‐climatic zones for the international exchange of data on the efficacy and crop safety of plant protection products. EPPO Bulletin. 2005;35(2):233–8.

[56] Tveito OE, Bjørdal I, Skjelvåg AO, Aune B. A GIS‐based agro‐ecological decision system based on gridded climatology. Meteorological Applications. 2005;12(1):57–68.

[57] Guy CL. Freezing tolerance of plants: current understanding and selected emerging concepts. Canadian Journal of Botany. 2003;81(12):1216–23.

[58] Kasuga M, Miura S, Shinozaki K, Yamaguchi-Shinozaki K. A combination of the Arabidopsis DREB1A gene and stress-inducible rd29A promoter improved drought-and low-temperature stress tolerance in tobacco by gene transfer. Plant and Cell Physiology. 2004;45(3):346–50. 15047884 10.1093/pcp/pch037

[59] Mizoi J, Shinozaki K, Yamaguchi-Shinozaki K. AP2/ERF family transcription factors in plant abiotic stress responses. Biochimica et Biophysica Acta—Gene Regulatory Mechanisms. 2012;1819(2):86–96. 10.1016/j.bbagrm.2011.08.004 21867785

[60] Yamaguchi-Shinozaki K, Shinozaki K. Organization of cis-acting regulatory elements in osmotic-and cold-stress-responsive promoters. Trends in Plant Science. 2005;10(2):88–94. doi: 10.1016/j.tplants.2004.12.012 15708346 10.1016/j.tplants.2004.12.012

[61] Albrecht V, Weinl S, Blazevic D, D'angelo C, Batistic O, Kolukisaoglu Ü, et al The calcium sensor CBL1 integrates plant responses to abiotic stresses. The Plant Journal. 2003;36(4):457–70. 14617077 10.1046/j.1365-313x.2003.01892.x

[62] Bacon CW. Abiotic stress tolerances (moisture, nutrients) and photosynthesis in endophyte-infected tall fescue. Agriculture, Ecosystems & Environment. 1993;44(1–4):123–41.

[63] Elmi A, West C. Endophyte infection effects on stomatal conductance, osmotic adjustment and drought recovery of tall fescue. New Phytologist. 1995;131(1):61–7. 10.1111/j.1469-8137.1995.tb03055.x 33863166

[64] Kannadan S, Rudgers J. Endophyte symbiosis benefits a rare grass under low water availability. Functional Ecology. 2008;22(4):706–13

[65] Thomas H, Evans C. Influence of drought and flowering on growth and water relations of perennial ryegrass populations. Annals of Applied Biology. 1990;116(2):371–82.

