# Supplementary material for: Intraspecific differences in long-term drought tolerance in perennial ryegrass
Source: PLoS One. 2018 Apr 4;13(4):e0194977. doi: 10.1371/journal.pone.0194977 (PMC5884532; doi:10.1371/journal.pone.0194977)
Supplement: S1 Appendix — (PDF) [file pone.0194977.s001.pdf]

S1 Appendix: Background information of germplasm accessions

Information about the background and origin of each accession was obtained from the National Plant Germplasm System (NPGS, USDA) and the New Zealand Margot Forde Forage Germplasm Centre (AgResearch). In addition to the nine accessions below, the perennial ryegrass cultivar 'Grasslands Impact' was used in this study. It is a late- flowering hybrid with improved winter growth and high autumn and summer yields under moist conditions [1].

A6889 ('Otago/Southland')

A6889 is an original germplasm, collected from Wendon, Southland, New Zealand on 1/4/1991.

A6932 ('Portugal')

A6932 was collected from Vila Pouca, Tras os Montes, North Portugal on 1/04/1991. This germplasm was collected by Forde & Easton, in the SW Europe collection, Forde & Easton ID 79.01. The seeds used were harvested from a planting of this accession in Gore, New Zealand on 1/1/1998.

A7798 ('France')

This accession was collected from France and is known as French Ecotypes ex Charmet 376. A7798 is recorded as an exchange germplasm. The seeds used were harvested in Palmerston North, New Zealand on 1/07/1992.

A14499 ('Turkey')

This accession was collected from North of Tabarka at the Turkish fort (Borj Massaoud) in Tunisia on 24/06/1994. It is labelled 'Turkey' in this study, to distinguish it from the other Tunisian accession (A15369, below). This accession from NPGS received the accession on 19/8/1994 and PI was assigned by 1997. The collection habitat was a roadway between sea and road and there was no grazing, with a slope of 6-10%. The climate is seasonally dry and the land was at sea level. The accession used is an increase of the 1999 collection and is an original germplasm. The seeds used were harvested from a planting in Palmerston North on 1/03/2006.

A14542 ('Italy')

This accession was collected from Campo Reggino, Umbria, Central Italy. The habitat of collection was a gateway of field and rough grazing land. This accession is known as increase of SW Europe Collection Forde & Easton ID 131.01. The seeds used were harvested in Palmerston North on 1/1/2006.

A15323 ('Algeria')

Accession A15323 was collected in Algeria and maintained by the Western Regional Plant Introduction (PI) Station. PI 231596 was received by the NPGS on 13/3/1956. Original plant inventory data shows the accession was from Berrouaghia, Algeria and the serial number given was 3228 with the date of entry 31/12/1956. The accession has been used in various studies, including Hulke *et al.* (2007). The

accession is known as PI 231586 Algeria increase and this accession is an exchange germplasm. The seeds used were harvested in Palmerston North on 26/1/2007 and observed to have poor heading.

A15334 ('Cyprus')

This accession was collected from Limassol, Cyprus and donated to NPGS on 10/01/1952. The PI was assigned in 1952. The accession was used in three different studies by Casler (1995), van Zijll de Jong *et al.* (2008) and Hulke *et al.* (2007). The screening of endophyte by Hulke *et al.* (2007) showed 2% of endophyte incidence out 52 tested plants. The accession is known as PI 198958, a Cyprus increase and is an exchange germplasm. The seeds were harvested in Palmerston North on 26/1/2007 and were observed to have poor heading.

A15369 ('Tunisia')

A15369 was collected near Skalba, 5 km west of Menzer Temine in Tunisia on 21/06/1994. The site of collection was dominated by tall grasses in a moist floodplain (Cheplick, 2007). NPGS received the accession on 19/8/1994 and the PI was assigned in 1997. The accession was studied by Cheplick (2007) and Hulke *et al.* (2007). It is called PI 598909, Tunisia increase and is an exchange germplasm. The seeds used were harvested in Palmerston North on 12/2/2007 and observed to have poor heading.

A17183 ('Norway')

This accession was collected in Bryne, Rogaland state, Norway and maintained by the Western Regional PI station. It was donated to NPGS in Wales, United Kingdom by the Welsh Plant breeding station on 03/09/1991. The PI was assigned in 1994. This material was used for winterhardiness and A17183 was ranked 5.5 in 2005 and 4.8 in 2006 by tiller survival whereas the best performing elite variety, Citation Fore had a rank of 3.2 and 2.7 [2]. These results indicate winterhardiness of accession A17183. This accession is an exchange germplasm and is an increase of PI577269 (A16719) Norway. The seeds used were harvested in Palmerston North on 17/1/2011.

References

1. Charlton J, Stewart A, editors. Pasture species and cultivars used in New Zealand-a list. Proceedings of the New Zealand Grassland Association; 1999;147-166
2. Hulke, Eric Watkins, Donald Wyse, Ehlke N. Winterhardiness and turf quality of accessions of perennial ryegrass (*Lolium perenne* L.) from public collections. Crop Science 2007;47:1596-608.
