# Supplementary material for: Intraspecific differences in long-term drought tolerance in perennial ryegrass
Source: PLoS One. 2018 Apr 4;13(4):e0194977. doi: 10.1371/journal.pone.0194977 (PMC5884532; doi:10.1371/journal.pone.0194977)
Supplement: S2 Appendix — (PDF) [file pone.0194977.s002.pdf]

S2 Appendix: Photographs of rhizotron

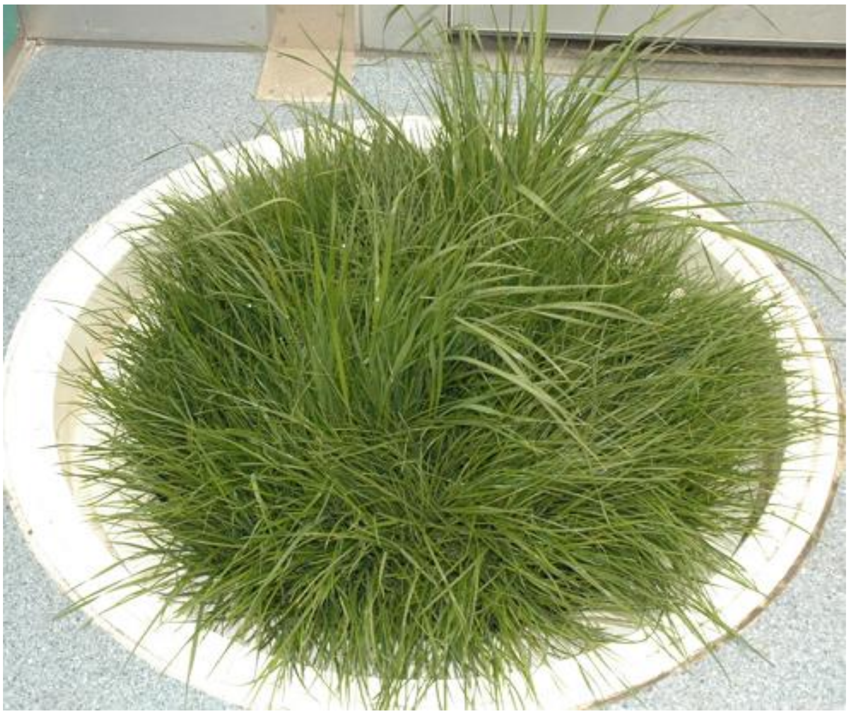

Figure A: well-established plants in rhizotron 1. The photograph was taken after the establishment phase on 12/09/2013 at the Biotron, Lincoln University.

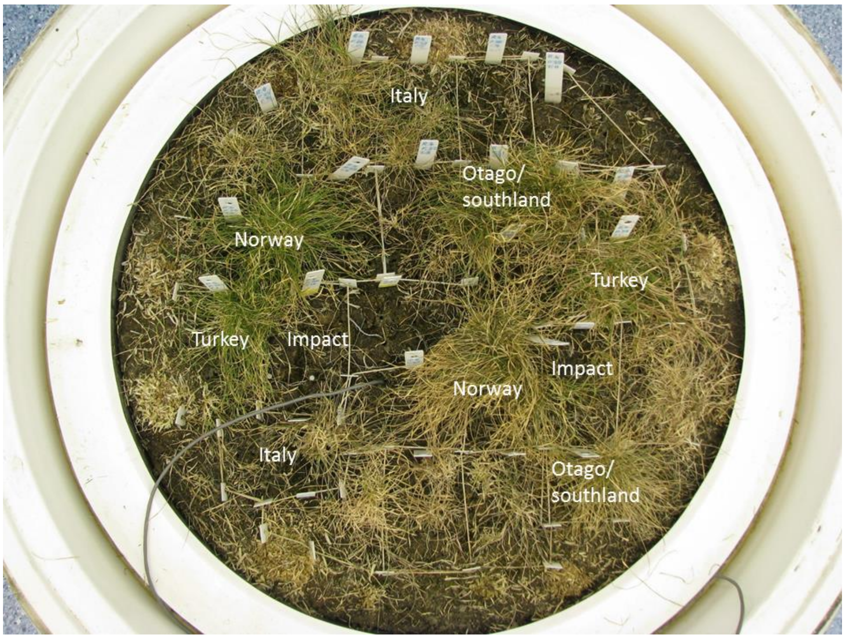

Figure B: Rhizotron 2 at the end of Cycle 6. Some of the high-yielding accessions and the reference cultivar 'Impact' are labelled to compare their responses to water deficit. The edge plants have been removed for clarity. The photograph was taken on 15/9/2014 at the Biotron, Lincoln University.
