# Supplementary material for: Intraspecific differences in long-term drought tolerance in perennial ryegrass
Source: PLoS One. 2018 Apr 4;13(4):e0194977. doi: 10.1371/journal.pone.0194977 (PMC5884532; doi:10.1371/journal.pone.0194977)
Supplement: S1 Table — (PDF) [file pone.0194977.s003.pdf]

Table S1: Summary of treatments and periods of each treatment under the study

Period	Start date	End date	Days of treatment
Establishment	5/08/2013	19/09/2013	45
Drought Cycle 1	20/09/2013	4/11/2013	45
Irrigation 1	5/11/2013	19/11/2013	14
Drought Cycle 2	20/11/2013	17/01/2014	58
Irrigation 2	22/01/2014	10/02/2014	19
Drought Cycle 3	11/02/2014	24/03/2014	41
Irrigation 3	25/03/2014	19/04/2014	25
Drought Cycle 4	20/04/2014	12/05/2014	22
Irrigation 4	13/05/2014	27/05/2014	14
Drought Cycle 5	28/05/2014	12/06/2014	15
Irrigation 5	13/06/2014	13/08/2014	61
Drought Cycle 6	14/08/2014	15/09/2014	32
