# Supplementary material for: Intraspecific differences in long-term drought tolerance in perennial ryegrass
Source: PLoS One. 2018 Apr 4;13(4):e0194977. doi: 10.1371/journal.pone.0194977 (PMC5884532; doi:10.1371/journal.pone.0194977)
Supplement: S2 Table — (PDF) [file pone.0194977.s004.pdf]

S2 Table: Endophyte detection

Results from endophyte screening from the seedlings generated from the available seeds.

Accessions	Number of seedlings	Positive	Negative	% of positive
‘Otago/Southland’	2	1	1	50
‘Norway’	11	0	11	0
‘France’	8	2	6	25
‘Cyprus’	15	0	15	0
‘Turkey’	3	0	3	0
‘Algeria’	11	0	11	0
“Italy’	12	0	12	0

NB: The analysis indicated the absence of endophyte symbiosis in the accessions used in the experiment. However, the number of seedlings available for screening were limited for some of the accessions such as ‘Turkey’ and ‘Otago/Southland’ (A14499 and A6889).
